# Orphan drugs’ clinical uncertainty and prices: Addressing allocative and technical inefficiencies in orphan drug reimbursement

**DOI:** 10.3389/fphar.2023.1074512

**Published:** 2023-01-26

**Authors:** Hans-Georg Eichler, Michael Kossmeier, Markus Zeitlinger, Brigitte Schwarzer-Daum

**Affiliations:** ^1^ Austrian federation of social insurances, Vienna, Austria; ^2^ Department of Clinical Pharmacology, Medical University of Vienna, Vienna, Austria

**Keywords:** orphan drugs, real world data, clinical uncertainty, drug prices, managed entry agreement, budget impact

## Abstract

Legislations incentivising orphan drug development and scientific advances have made orphan drugs pharma’s high-end favourite for the past two decades. Currently, around 50% of new marketing authorizations are for orphan drugs. For third-party healthcare payers (“payers”) the rise of orphan drugs presents new challenges, including a high degree of uncertainty around clinical benefits and harms, a moderate effect size (for many orphan drugs), and a high price tag. The association of high clinical uncertainty and moderate effect sizes is not surprising in small target populations but in combination with high prices creates the risk of allocative and technical inefficiencies for payers. We here discuss and illustrate these risks. A combination of policies is needed for mitigation of allocative inefficiency: while there may be a rationale for higher prices for orphan than non-orphan drugs, a focus of pricing and reimbursement negotiations should include considerations of product profitability and of the consequences of orphan drug costs on the distribution inequality of medication costs for individual insured persons, coupled to knowledge generation from reimbursement contracts covering high-price orphan drugs that would benefit the wider patient community. Performance-based managed entry agreements could help to de-risk the economic consequences of clinical uncertainty and to mitigate technical inefficiency.

## Introduction

During the age of the “blockbusters” (Trusheim et al., 2007), drugs for rare (also known as orphan) diseases took a back seat in the development portfolios of most pharmaceutical companies. However, legislations enacted in several jurisdictions to incentive orphan drug development and scientific advances such as the field of genetics have converged to make orphan drugs pharma’s high-end favourite for the past two decades.

Currently, around 50% of new marketing authorizations are for drugs indicated for orphan conditions. This shift in focus is a welcome success for some patients but the majority of orphan diseases remains an area of high unmet medical need, with around 90% of rare diseases having no approved treatment today (Pearson et al., 2022). There is reasonable hope and expectation that the near future will see a raft of new orphan drugs come to market, both for hitherto untreatable conditions and novel treatment options for conditions for which treatments already exist.

From a drug developer’s perspective, the rationale for banking on orphan drugs is simple and compelling: so called “niche buster drugs”, i.e., treatments for small target populations but with a high price tag can generate similar revenues as “blockbuster drugs”, i.e., those for large target populations but with a comparatively lower price tag (Trusheim et al., 2007).

For third-party healthcare payers (“payers”), the rise of orphan drugs is welcome because payers can now, for the first time, offer treatments to some of their insured patients, but niche busters also create new headaches and challenges for them, including the dramatic rise over the past years of the fraction of total dug budgets consumed by orphan drugs (discussed in detail below).

In this article we describe the challenges from a payer’s perspective that come with a shift from the blockbuster to the niche buster paradigm, focusing on orphan drugs; we discuss the risks of allocative and technical inefficiencies associated with paying for highly expensive orphan treatments. To conclude, we propose solutions to help address these potential risks.

We will use the term “orphan” as per current EU regulation which defines “orphan conditions” as a prevalence of not more than five in 10,000 persons in the EU (Official Journal of the European Communities, 2000).

### How is reimbursement for orphan drugs different from non-orphan drug reimbursement?

The first challenge for payers is moderate (or “incremental”) effect sizes. Precision (oncology) medicines and novel pharmaceutical platform technologies like gene therapies or RNA technologies have come with a promise of dramatic “breakthrough” effects for patients with orphan diseases.

Some therapies have indeed delivered on that promise. Consider the case of adenosine deaminase (ADA)-deficient severe combined immunodeficiency (SCID), a very rare congenital disorder of the immune system. If left untreated, ADA-SCID is typically fatal within a child’s first year of life. In 2016, Strimvelis™, an *ex vivo* hematopoietic stem cell (HSC) gene therapy designed to correct the underlying gene defect, received marketing authorisation in the EU (Aiuti et al., 2017). The authorisation was based on data collected from a total of 18 ADA-SCID children, with a median follow-up of about 7 years; survival was 100% at the time of approval. This impressive “breakthrough effect” was recently confirmed in a larger series of patients (Kohn DB et al., 2021).

Regrettably, the effect size seen with Strimvelis was the exception rather than a rule when it comes to novel orphan drugs. More often we are faced, e.g. in rare cancers, with a “[p]rogression-free survival gain of 4 months between treated and non-treated, a response rate of 65%, but no survival benefit, single-arm studies with challenges to interpret the results….” (Leufkens et al., 2022). Duchenne muscular dystrophy (DMD) affords another example: several therapeutic strategies have been investigated and a small number of products have received marketing authorisation; while the effect sizes are non-null, they are far away from being a cure (Yao S et al., 2021).

We hasten to emphasise that such incremental benefits should be neither belittled nor rejected. Patients with a high unmet medical need will understandably desire access to new therapeutic option with small or marginal effects–when the alternative is null effect. Science progresses in small steps and many small steps may add up to a large step. In some instances, the full potential of new agents is only realised after prolonged on-market learning of how to best use, dose, and judiciously combine them with other agents to achieve supra-additive effects; this has been shown, for example, with the combination of BRAF and MEK inhibitors which improved overall survival in melanoma BRAF mutant melanoma (Lopez and Banerji, 2017). It follows that a (moderate) effect size apparent at the time of marketing authorisation and initial drug reimbursement is not necessarily fixed over time. Finally, some desperate patients or parents may wish to buy time: “If this treatment extends the life of my child by only a few months, perhaps something better comes up within these months.” We explicitly reject the notion that small or moderate effect sizes “are not worth it” and that development and authorisation of incremental new therapies should be discouraged.

The second challenge is uncertainty about net clinical benefit at the time of marketing authorisation which results from uncertainty about both benefits and risks of new orphan drugs. Traditionally, payers focused mostly on the benefit side, i.e., clinical effect size. In the following we will therefore continue to focus on effect size.

Uncertainty is caused to no small extent by the observed moderate effect sizes of new orphan drugs discussed above. To understand the interaction between effect size of drugs and uncertainty, we recall a simple statistical fact: small effects are harder to demonstrate and quantify than large ones. This is not a new insight but it comes to the fore as we see a growing number of drugs for rare and very rare conditions coming to market.

Table 1 intends to further elaborate the relationship between effect size, size of target patient population, and level of evidence about new products coming to market.

**TABLE 1:** Relationship between the size of clinical effect, size of target population, feasibility of different study types, and credibility of results.

Type of evidence generation (study type) that may be feasible/unfeasible and/or credible	Large target population	Small target population
Large effect size	RCT: even small studies are likely to be adequately powered, due to large effect size; sufficient N of clinical trial patients available → RCTs feasible, results credible	RCT: even small studies are likely to be adequately powered, due to large effect size; sufficient N of clinical trial patients likely available, even in (some) rare diseases → RCTs likely feasible, results credible
	RWD studies: extant RWD likely available; effect size expected to be much larger than risk of bias or chance findings, → RWD studies feasible and results likely credible	RWD studies: extant RWD likely available, e.g., historical control cohorts; effect size expected to be much larger than risk of bias or chance findings, → RWD studies feasible and results likely credible
Small effect size	RCT: due to small effect size, trials need to be large to be adequately powered; but sufficient N of clinical trial patients available → RCTs feasible, results credible	RCT: due to small effect size, trials need to be large to be adequately powered; sufficient N of clinical trial patients not available, perceived ethical concerns may reduce patients’ willingness to be randomised → RCTs likely unfeasible, no results available
	RWD studies: extant RWD most likely available (e.g., historical control cohorts); but due to small effect size, studies are expected to be highly susceptible to risk of bias or chance findings → RWD studies feasible but may not be credible	RWD studies: extant RWD may or may not be available (e.g., historical control cohorts); due to small effect size, studies are expected to be highly susceptible to risk of bias or chance findings → RWD studies feasible might be feasiblebut may not be credible

N = numbers; RCT, randomised controlled trials; RWD, real world data.

Note that the variables “target population” and “effect size” are in fact continuous variables. Nonetheless, we dichotomised both variables (large *versus* small) for ease of explanation and to extract generalizable learnings. The traffic light colour scheme illustrates the feasibility of generating robust evidence about drug effects in each quadrant.

The focus of attention is on the lower right-hand quadrant: orphan drugs for rare or very rare conditions with small to moderate effect sizes. In these situations, usually coupled with a high unmet medical need, the conduct of adequately powered randomised controlled trials (RCTs) may not be feasible because of small numbers of patients available for recruitment and, not infrequently, because of real or perceived ethical issues precluding randomisation or unwillingness on the part of patients to be randomised. At the same time, observational studies, e.g., by relying on external comparator groups based on extant real world data (RWD) might be ethically and technically feasible but RWD studies are difficult to interpret in the context of small therapeutic effect sizes due to the risk of bias. The result may be “unavoidable uncertainty” about the effects of many drugs that fall into this quadrant.

Note that the variables “target population” and “effect size” are of course continuous variables. Nonetheless, we dichotomised both variables in Table 1 to present our argument and for ease of explanation. The focus of attention is on the lower right-hand quadrant: orphan drugs for rare or very rare conditions with small to moderate effect sizes. In these situations, usually coupled with a high unmet medical need, the conduct of adequately powered randomised controlled trials (RCTs) may not be feasible because of small numbers of patients available for recruitment and, not infrequently, because of real or perceived ethical issues precluding randomisation or unwillingness on the part of patients to be randomised. At the same time, observational studies, e.g., by relying on external comparator groups based on extant real world data (RWD) might be technically feasible but RWD studies are difficult to interpret in the context of small therapeutic effect sizes due to the risk of bias and sometimes due to change in patients population over time (e.g., less severe patients due to earlier diagnosis).

The direct consequence for payers of having to take decisions on products in the lower right-hand quadrant (Table 1) is “unavoidable uncertainty” about the clinical performance (benefits and harms) of such products. We emphasize that the term unavoidable uncertainty should not serve as an excuse for companies to not conduct trials that might be feasible. However, stakeholders, including payers, need to accept that some research questions, including on (comparative) effectiveness, cannot be answered, at least at the time of marketing authorisation.

The indirect consequence of the growing number of products that sit in the lower right-hand quadrant (Table 1) is that payers have to not only pay high prices for (probably) small effects but also under conditions of considerable uncertainty as to which or how many patients will benefit at all, how much, for how long, or how to best select treatment-eligible patients. The high levels of uncertainty will render traditional health technology assessment (HTA) methods meaningless, at least at the time of initial pricing and reimbursement (P&R) negotiations when much needed information is not available (Pearson et al., 2022).

The obvious third challenge for payers is drug prices: the issue has been much publicized, with news in the general media reporting on “million Dollar [or Euro] drug costs per patient” (Thomas and Abelson, 2019). Price tags for one-off gene therapies are usually above the hundred thousand € or $ mark and the one million per patient threshold has repeatedly been crossed. Similar per-patient (per-year) prices are currently charged for biological or small molecule drugs that need repeat administration for the treatment of chronic orphan conditions (Thomas and Abelson, 2019; Pearson et al., 2022).

In the past, some payers have taken the view that such prices can be absorbed by healthcare systems in wealthy economies; since the absolute number of patients for whom these prices were paid was small, the overall budget impact was manageable. However, as the number of orphan indications (e.g., in oncology due to the identification of genetically-specified subpopulations), the number of high-price products, and the number of treatment-eligible patients keeps growing, there is a more recent perception that the budget impact can no longer be ignored and the system may reach a breaking point (Villa F et al., 2022). We do not expect healthcare systems to reach breaking point as a result of growing prices; healthcare budgets have been slowly rising in most high-income countries and drugs with ever higher list prices are being funded. However, budgets for healthcare cannot be easily increased in the short term and we expect a growing number of high price orphan drugs for which funding will be declined or restricted to much smaller (sub-)populations than the benefit-risk profile suggests.

High prices have already plagued the launch of the first generation of Advanced Therapy Medicinal Products (ATMPs, i.e., gene and cell therapies): at the time of writing, 7 ATMPs (4 of which with orphan status) authorised in the EU were taken off the EU-market, often as a result of failed P&R negotiations. (EMA, 2022). This undesirable outcome impedes access for patients to potentially useful treatments. Paradoxically, the price issue may also disincentivise some drug development programs by showing that treatments of (ultra)rare disorders remain at substantial risk of commercial failure, even when they receive a marketing authorisation.

An additional financial concern for payers is the shift of the cost of evidence generation from manufacturers to payers. RCTs have typically been the main cost factor in the development of new drugs, and they are usually conducted before approval at the expense of the sponsor, allowing HTA organisations and payers to estimate clinical benefits on a solid ground when negotiating a price. When marketing authorisation of (orphan) drugs is granted on the basis of limited clinical evidence, a substantial part of evidence generation needs to happen in the post-authorisation phase and is based on RWD that may have to be (co-)financed by payers who need to establish infrastructure and processes for data collection. At the same time, patent periods are extended and profits remain with the manufacturer.

The unwelcome combination of incremental and often uncertain clinical benefits with breakthrough prices presents a toxic problem for payers: the resulting low cost-effectiveness of such products aggravates the “payers’ dilemma” of having to ensure access to treatments especially for patients with high unmet medical need, while at the same time ensuring efficient and equitable use of limited resources. The combination of these challenges is the root cause for payers’ risk of allocative and technical inefficiencies in their decision-making.

### The risks of allocative and technical inefficiencies

Economic efficiency in healthcare delivery implies that society makes choices which maximise the health outcomes gained from the limited resources allocated to healthcare. Inefficiency exists when resources could be reallocated in a way which would increase the health outcomes produced (Palmer and Torgerson, 1999). Economists describe different types of efficiencies such as technical, productive, and allocative efficiencies. (For a definition and in-depth discussion of types of efficiency, please refer to Palmer and Torgerson, 1999).

*Technical efficiency* refers to the relation between resources and health outcome. A technically efficient position is achieved when the maximum possible improvement in outcome is obtained from a set of resource inputs. An intervention is technically inefficient if the same (or greater) outcome could be produced with less of one type of input, e.g., a given orphan drug in a setting of no or unsatisfactory alternative treatments.

*Productive efficiency* enables assessment of the relative value for money of interventions with directly comparable outcomes. However, it cannot address the impact of reallocating resources at a broader level, across disease areas or patient groups, because the health outcomes are incommensurate (Palmer and Torgerson, 1999).

*Allocative efficiency* takes account not only of the technical and productive efficiencies with which healthcare resources are used but also the efficiency with which outcomes are distributed among the community. Such a societal perspective is rooted in welfare economics and has implications for the definition of opportunity costs (Palmer and Torgerson, 1999); it also touches on the issue of fairness of distribution of resources.

Practical experience shows that, in day-to-day P&R negotiations for orphan drugs, considerations of productive efficiency may occasionally come up (e.g., for different treatments for spinal muscular atrophy) but are hampered by uncertainty about (relative) value. Most orphan drug P&R negotiations are individual, one-off reimbursement decisions for indications with no or unsatisfactory treatment options. We will therefore limit our discussion to technical and allocative efficiencies and subsume issues of productive efficiency under these categories.

To elucidate the risks of inefficiencies in the context of orphan drug reimbursement, we first consider the hypothetical scenario of a newly authorised orphan drug for which near-complete information is available on optimal selection of treatment-eligible patients, effect size (at least on the average effect size for the defined target population), dose, duration of treatment, etc. Here, the clinical benefit and economic value can be assessed ex ante, by way of HTA, and the treatment can be used to maximum effect. Since the right drug can be given at the right dose to the right patient at the right time, (most) patients are expected to experience the expected clinical benefit and technical inefficiency is not a major risk. However, if the price of the drug is extremely high, the risk of allocative inefficiency remains. Is it efficient to allocate a high fraction of the available drug budget to a small number of orphan patients when the same amount of budget could perhaps produce more societal benefit if reallocated to serve a larger patient group? Is it fair to invest so much money at so few in the confines of a solidarity-based insurance system? The question brings us back to the payers’ dilemma. Note that niche buster drugs create more asymmetry on the payer side: blockbuster drugs distribute healthcare funds more evenly across the insured population than do niche busters.

We acknowledge that these questions will take most decision-makers, including payers, healthcare providers, and indeed patients, outside their comfort zone. However, the question is already being debated, either explicitly or—more often—implicitly, and will come to the fore with the advent of more high-price orphan drugs.

Leufkens et al. (2022) have recently sketched future scenarios where one might expect that “some patient advocates still March for enabling everything that is possible…,” while others argue that “[i]nvesting massive resources in the few contrasts with the health needs of the many, backed by business models that do not appear to be helpful in shifting investments to priority medicines and public health needs … ”; consequently, “[m]edicines for rare diseases, pharma’s high-end favourite for about two decades, receive … critical exposure” (Leufkens et al., 2022). There is now a real risk of loss of solidarity among the community (Leufkens et al., 2022) of insured persons in the face of ultra-high costs for a few (Thomas and Abelson, 2019).

We would consider such a future scenario undesirable, both for patients with orphan disease, and for society at large, for reasons outlined below. We argue that it is better to explicitly address the issue of allocative efficiency of orphan drug reimbursement and ask what is a justifiable price in a structured and transparent way rather than attempt to muddle through.

Minimising allocative inefficiency is only one of the payers’ concerns. In addition, they need to tackle the risk of technical inefficiency when negotiating prices and coverage for orphan drugs.

We recall that the hypothetical scenario of near-complete information is far from reality and most new orphan drugs come with considerable—and often unavoidable—uncertainty about the clinical effect size. Uncertainty may result in a situation where payers spend scarce resources on treatments for patients who will accrue no or minimal clinical benefit which, in turn, translates into technical inefficiency, i.e., expenses without realising the expected return.

How are orphan drugs different from non-orphans in regard of technical inefficiency? Payers have always found themselves paying for some *individual* patients who experience little or no benefit from the treatment, even for non-orphan or blockbuster drugs. However, in the presence of high-quality RCT evidence, the *average* clinical benefit and effect size for the treatment-eligible population can be quantified ex ante and payers can base their P&R negotiations on the expected average clinical outcome for a group of insured patients, including non-responders. While the power of RCT results to predict treatment success under conditions of everyday clinical practice is less than perfect (Eichler et al., 2022), available RCT evidence is expected to at least mitigate, if not eliminate, technical inefficiency. Contrast this to a situation where clinical evidence about a novel orphan product is limited to a small, uncontrolled case series of patients, often coupled to relatively short observation periods in the clinical trial setting. Here, not even an average effect size can be predicted. Payers are flying blind when trying to predict value and the risk of technical inefficiency due to lack of effect is high.

To conclude, the risks of allocative and technical inefficiencies are much higher in the context of orphan than non-orphan drug reimbursement, presenting payers with a new challenge that will require new solutions.

### How can we mitigate allocative and technical inefficiencies?

#### Allocative inefficiency

To address the issue of allocative (in)efficiency we start from the obvious premise that solidarity-based health insurance schemes are designed to enable unequal payments to members who pay in (more or less) equitable premiums. Payments to individual insured members should be dictated by health needs which are mostly unforeseeable. However, this is not the same as giving systematic preference to a particular subgroup of the insured population, i.e., patients with orphan diseases, by, for example, allowing higher cost-effectiveness thresholds when paying for orphan compared to non-orphan drugs.

The debate over systematically higher prices and/or cost-effectiveness thresholds for orphan drugs has been going on for at least a decade. Surveys of the general public seemed to suggest that there is no willingness to pay a premium for rarity (Berdud et al., 2020), and some health economists take the view that cost-effectiveness thresholds should not shift systematically solely on the basis of rarity, as such shifts threaten the goals of health equity (Pearson et al., 2022).

If executed without compromises, an “equitable” policy would lead to most orphan drugs being denied reimbursement (Berdud et al., 2020) but, in reality, many orphan drugs are currently being reimbursed in many high-income jurisdictions. The custom of accepting higher prices for (ultra-)rare disease products suggests a societal willingness to pay more for these products. Some countries have established separate procedures for value assessment (Villa F et al., 2022) and funding of therapies for ultra-orphan populations that may include higher thresholds (Pearson et al., 2022).

Is there an underpinning in societal expressed preferences or legislation of giving allocative preference to patients with orphan diseases?

The primary author of the US orphan drug act (ODA), the politician Henry Waxman, stated in 1986, “The [ODA] is meant to demonstrate that society puts a higher value on helping victims of rare disease than does the pharmaceutical marketplace” (quoted in Pearson et al., 2022).

We argue that this sentiment is implicitly expressed in the EU orphan legislation (Official Journal of the European Communities, 2000) which states that “…patients suffering from rare conditions should be entitled to the same quality of treatment as other patients” and, acknowledging the need to incentivise orphan drug development, establishes a range of benefits, including 10 years market exclusivity for authorised orphan drugs products (though some restrictions may apply). It would be illogical or naïve to, on the one hand, declare orphan drugs a societal priority and expect equal quality of treatment, while, on the other hand, expect equal expenditure in the presence of an artificial monopoly created to correct market failure.

We argue that the acceptance of higher prices (or higher cost-effectiveness thresholds where applicable in a given payer system) is built into the orphan drug legislation, is compatible with the aim of offering orphan patients much-needed therapy, and does not *per se* constitute allocative inefficiency or lack of fairness. However, accepting this premise does not justify *any* price level and raises the crucial question “how high is too high a cost?”

In the absence of an obvious and uncontested answer, we agree with the reasoning of (Berdud et al., 2020), that even when “societal decision-makers may be willing to pay above their standard value-based price to make treatments for some orphan diseases available, they would still need a benchmark for use in price negotiations.”

We here describe three types of considerations or benchmarks for payers to take into account when addressing the dilemma of allowing for higher orphan drug prices while mitigating allocative inefficiency.

(i) *Avoiding unjustified profitability:* A supplier-oriented guide for setting prices was proposed by (Berdud et al., 2020): they argue that a price paid for a given orphan product should “ensure that the manufacturers of orphan drugs do not make higher profits than manufacturers of drugs for non-orphan conditions.” We support this notion on the grounds that it reflects the spirit of the orphan drug legislation which states that the period of market exclusivity may be reduced “…where it is shown on the basis of available evidence that the product is sufficiently profitable not to justify maintenance of market exclusivity” (Official Journal of the European Communities, 2000). Orphan legislation is intended to correct market failure, not to support the marketing of super-lucrative products for individual companies.

In order to establish a maximum price range based on this proposition, Berdud et al. (2020) have examined how the standard incremental cost-per-quality adjusted life year (QALY) cost-effectiveness threshold (CET) in the United Kingdom would need to be adjusted to reflect typical differences between orphan and non-orphan products. The key differences they considered included: the costs of R&D (around 20%–25% of orphans to non-orphan, mostly due to smaller clinical trial size and other drug lifecycle costs), difference in the overall development success rate (defined in terms of the proportion of drugs obtaining a market authorization, which is another key driver of overall R&D cost; this was 34.6% for orphans and 5.9% for non-orphans), and in the size of the expected treatment population (non-orphan rounded average: 100 per 50,000, orphan mid-point population: 12.5 per 50,000, ultra-rare cut-off population: 1 per 50,000).

Acknowledging unavoidable limitations of their methodology and model inputs, they estimate that for orphan populations (population size 12.5 to 25 per 50,000) the incremental CET should be no more than 2–4 times higher than the standard CET (around UKP 20,000), while for ultra-orphan populations (prevalence around 1 per 50,000) it should be no more than around 50 times higher.

Different CETs cannot be directly translated into price differentials and many healthcare payers do not even make use of CETs. Yet, it is interesting to note that (Pearson et al., 2022) have recently quoted that: “In 2017, the average annual cost of orphan drugs at launch was 25 times higher than the annual cost of treatment for non-orphan drugs […]. Another analysis found that among the top 100 drugs by U.S. sales, the average cost of treatment for orphan drugs is 4.5 times that of non-orphan drugs […].” (Note that there is no differentiation between orphan and ultra-orphan drugs).

Berdud et al. (2020) emphasise that their results on increased CETs do not indicate what society *should* be prepared to pay for an orphan drug, but should be viewed as one way of determining the *maximum* price society should be willing to pay to ensure a reasonable rate of return (Berdud et al., 2020). We concur that third party payers should reject paying orphan drug prices that would result in higher than the industry average returns on investments, in order to fulfil their statutory obligation to ensure allocative efficiency and fairness. It follows that payers should request, and indeed insist, on obtaining full financial transparency from a manufacturer when there is reason to believe that an asking price would result in unjustified returns (as discussed above). We are aware that manufacturers may be reluctant to disclose such information and even if it is forthcoming, the data may be difficult to interpret. This is because considerations of the manufacturer’s profitability need to account not just for cost of goods (which may be relatively high for some biologicals and ATMPs) and the development costs of the product in question but also for failed development programs and any basic research into the disease mechanism that the manufacturer may have undertaken.

These caveats notwithstanding, we expect that bringing orphan drug returns into the P&R discussion will help mitigate excessive allocative inefficiency. Moreover, we are hopeful that this line of argument would be supported by concerned patient organisations.

(ii) *Considerations of fairness of spending:* We here propose another consideration for P&R negotiations that is directly based on fairness and may help mitigate allocative inefficiency. Our starting point is the distribution of the annual per patient drug expenditure in a given health insurance system.

**FIGURE 1:**
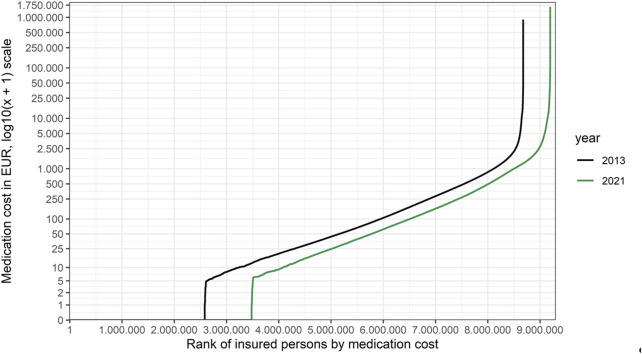
Medication costs for individual insured persons in the Austrian public statutory health insurance in 2021 *versus* 2013. The costs of drug treatments reimbursed for all individual, insured patients incurred by the Austrian public statutory health insurance were retrieved from the administrative database of the Austrian Federation of Social Insurances. In total, 99.9% of the Austrian population is covered by the Austrian public statutory health insurance and hence included in the database (Citation: Austrian Federation of Social Insurances (2021) Jahresbericht der Österreichischen Sozialversicherung. URL https://www.sozialversicherung.at/cdscontent/load?contentid=10008.747502&version=1621948595). The administrative database used holds a complete (pseudonymised) listing of all prescription drugs reimbursed (€ spent, per person, per day). For each year (2021 and 2013) all drug costs per person incurred between 01 January and 31. December were added. Costs are not inflation-adjusted. All analyses were performed using the statistical software R (version 4.2.0). (Citation: R Core Team (2022). R: A language and environment for statistical computing. R Foundation for Statistical Computing, Vienna, Austria. URL https://www.R-project.org/). Costs are shown for all insured persons (around 9.2 million persons in 2021, and around 8.7 million in 2013), ranked from lowest (left) to highest payments per person (right). Note that the y-axis is logarithmic (log10 (x+1)). The shape of the curves is explained by the fact that zero medication costs were incurred for around three million persons in both years. For interpretation of the data, please see main text.

Using the Austrian public social insurance organisations as an example of a comprehensive, affluent and developed solidarity-based public healthcare system, we present in Figure 1 the distributions across the insured population of the drug costs in the outpatient sector incurred by the Austrian public statutory health insurance. We compare costs in the year 2021 (the most recent year for which data are available) with the year 2013 (the earliest year for which such granular data are available; data were not inflation-adjusted) in order to have a long enough time span to assess long-term trends.

When interpreting the data in Figure 1, there are three important caveats to be aware of: i) some (prescription) drug costs are below the level of co-pay (€5.90 excl. VAT per pack of medication in €2021 and €4.80 excl. VAT per pack of medication in 2013) and will therefore be paid out-of-pocket by the insured persons (except for insured persons exempt of paying the prescription fee). These medication costs will appear as zero cost to the healthcare system and explain the unexpected bump in the curves in Figure 1 (near the 3 million person mark on the x-axis); ii) drugs (orphan or non-orphan) that are administered in the hospital setting are not captured in those numbers as the drug cost is incurred by the hospitals and reimbursed by the social insurances as a part of bundled payments. These are mostly biologics or ATMPs. Sometimes, with development of oral or subcutaneous formulations they become self-administered and therefore come into the drug budget; iii) medication costs per insured person are based on list prices paid by the social insurances, i.e., negotiated rebates are not taken into account due to confidentiality agreements. However, these shortcomings do not affect the observed time trends and overall conclusions on distribution of medication costs.

Inspection of the curves shows that the distribution of medication costs is extremely skewed. This is not unexpected but we note that the median costs decreased from €25.9 in €2013 to €17.0 in 2021 (even though prices were not inflation-adjusted), a consequence of the increasing number of prescriptions belonging to the group of drugs with a price below the level of co-pay. On the other hand, costs at the 99.5% quantile increased from €6,901.9 in €2013 to €10,803.0 in 2021, with the highest per patient cost rising from €0.9 M in €2013 to €1.6 M in 2021. Hence, the ratio of 99.5% quantile/median (= 50% quantile) cost had increased from 267.0 to 635.5, indicating a substantial shift of spending to the right over the past 8 years. Consistently, the number of patients with medication costs exceeding €100.000 per year has increased sevenfold from 217 patients in the year 2013 (of which none exceeded €1.000.000) to 1,586 patients in the year 2021 (of which three exceeded €1.000.000).

**FIGURE 2:**
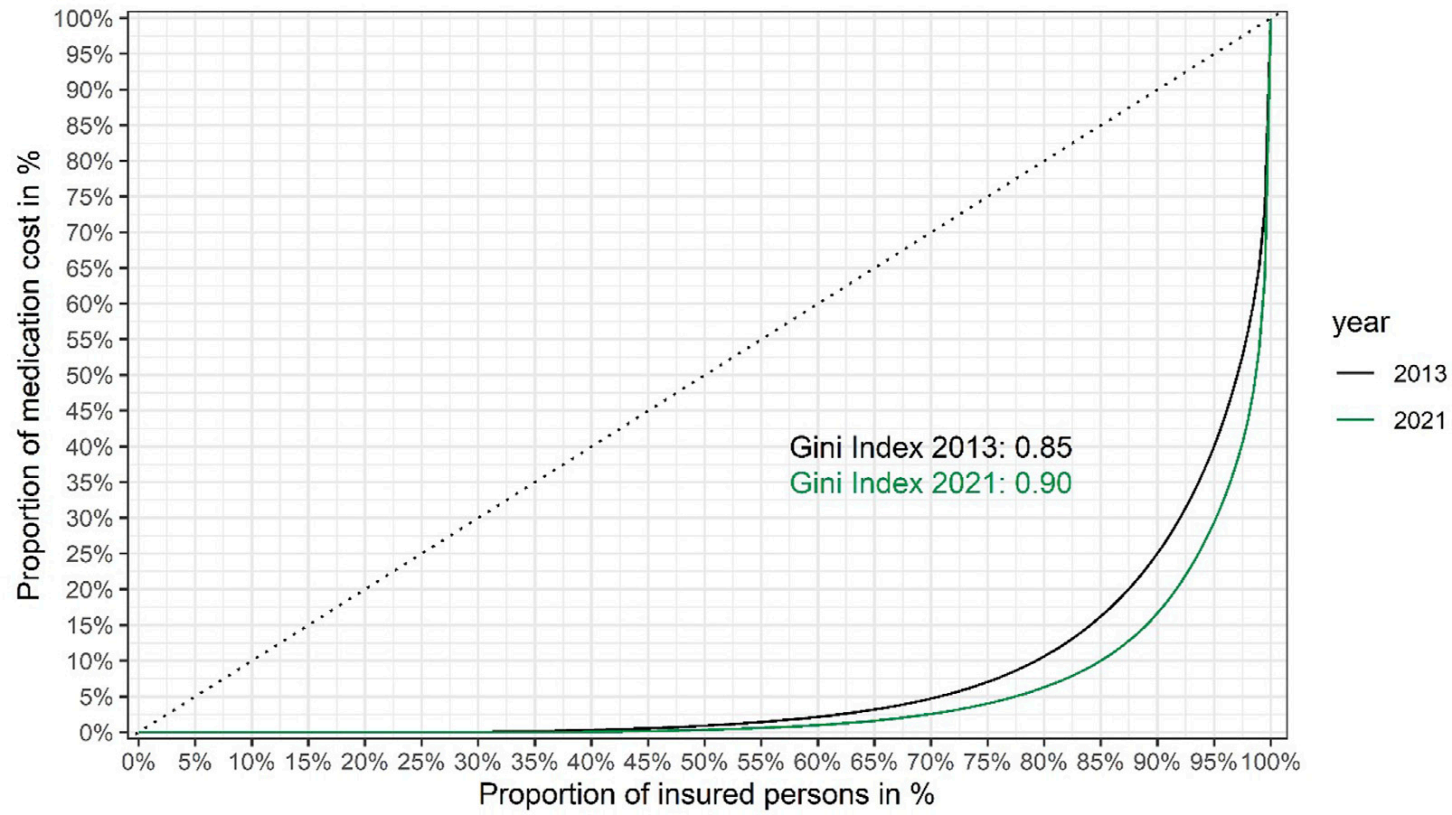
Lorenz curves showing inequality of medication cost per insured person in the Austrian public statutory health insurance in 2021 *versus* 2013. Insured persons are ranked from left to right based on their share of the total cost of medication to the Austrian public statutory health insurance (the total number of insured persons was around 9.2 million in 2021, *versus* around 8.7 million in 2013). The cumulative proportion of the total healthcare costs for each quantile are shown on the y-axis. The dashed diagonal line indicates cumulative costs in a hypothetical, completely equal scenario (i.e., where each insured person would consume the exact same share of the total medication costs). The Lorenz curves (solid lines) show the observed cumulative costs in 2021 and 2013, respectively. The Gini coefficients of inequality (0.90 and 0.85 in 2021 and 2013, respectively) were calculated from the respective Lorenz curves as twice the area between the diagonal line of complete equality and the Lorenz curve of the observed distribution of medication costs. Gini coefficients were calculated using the R package ineq. (Citation: Zeileis A. (2014). ineq: Measuring Inequality, Concentration, and Poverty. R package version 0.2–13, <https://CRAN.R-project.org/package=ineq>).

Based on the data shown in Figure 1, we also present Lorenz curves of the cumulative fraction of the total drug budget consumed by different groups (quantiles) of insured persons (Figure 2).

From the Lorenz curves we calculated the Gini coefficient (also known as Gini index) (Office for National Statistics website), a widely used summary measure of inequality in the distribution of income or cost. (The lower its value, the more equally income or cost is distributed.) Figure 2 shows that between 2013 and 2021 the Gini coefficient has risen from 0.85 to 0.90, indicating that inequality of medication spending is on the rise.

The figures discussed above are about total drug costs; we now focus on orphan drug spending: in the year 2013, 3.8% of the total drug budget (of €2.63 billion) was spent on orphan drugs for 0.05% of the insured population; by 2021 that fraction had risen to 8.0% (of €3.70 billion), for 0.07% of the insured population. The share of total drug costs for persons receiving orphan drugs (i.e., the cost of orphan and non-orphan drugs reimbursed for those persons) had increased from 4.5% to 9.2%, respectively. Our numbers are in broad agreement with those of the Italian National Health Service; Villa F et al. (2022) reported that “In 2020 in Italy … [t]he orphan drugs’ spending, being 1.4 billion euro, has represented a 6.0% share of the total public pharmaceutical expenditure.” We conclude that the old adage that “orphan drugs are expensive but the budget impact is minimal” is no longer tenable—the budget impact of orphan drugs is important and rising rapidly.

However, a high budget impact does not necessarily imply that orphan drugs increase inequality of spending. We calculated the Gini coefficient of medication costs in 2021, excluding all persons for whom costs for orphan drugs were incurred: the Gini coefficient was only reduced to 0.89, compared to 0.90 for the total population (see Figure 2, the curve excluding costs for persons receiving orphan drugs is not shown for clarity of presentation, as the two curves are nearly overlapping). The surprisingly small difference is likely explained by the small number of orphan patients (around 6,500). The key conclusion is that while orphan drugs add to the inequality of medication costs, their contribution is relatively small and spending on non-orphan drugs (such as non-orphan cancer drugs) is also highly unequal.

It is worth reminding that this conclusion and the data discussed above relate merely to inequality of spending, not necessarily to the level of allocative (in-) efficiency as they show cost per person, rather than cost per unit benefit. Note that the concept of benefit may also include societal benefit, e.g., when younger patients are able to go back to work and therefore provide money to the Social Security system instead of requiring financial support. Yet, in the absence of reliable information on clinical and societal benefit, drug price and anticipated cost per patient may be the only robust information available to payers at the time of P&R negotiations.

How could these findings guide payers to address allocative inefficiency of orphan drug reimbursement? We do not advocate for a per-person spending threshold (i.e., a maximum allowable fraction of a given budget for one individual patient). Not only would a threshold be difficult to implement politically and socially but it might defeat the purpose of allocative efficiency in case a highly expensive products delivers exceptional clinical benefit (e.g., a truly curative gene therapy for a lethal disease in new-borns).

However, we submit that any payer could estimate the position of the anticipated cost/person/year on a curve as shown in Figure 1, at the time of initial price negotiations when confronted with the asking price of the product. We advocate that any orphan product priced to sit above a given threshold (e.g., the 99.5% or 99.8% quantile) should automatically trigger two actions: first, a mandatory request of full transparency about anticipated profitability of the product, as discussed above. Second, mandatory implementation of special payment scheme (see below). The second action need not be limited to orphan drugs.

Including the cost distribution curve (as in Figure 1) to guide P&R negotiations makes economic sense, by focusing the payers’ efforts on the very high end products, as well as ethical sense. A health insurance system characterised by a Gini coefficient of 0.0 (meaning that every insured person gets exactly the same from the system) would be pointless, but a Gini coefficient approaching 1.0 (meaning that the entire budget goes to one individual) is also not compatible with a solidarity-based insurance system. The sweet spot has to be within a range somewhere between these two extremes but we are not aware of publicly available guidance or examples of where it should be located. While we cannot recommend an “ideal” Gini coefficient, these authors take the view that the sweet spot for the Gini coefficient for healthcare (or drug) expenditure may be higher than frequently published Gini coefficients for national income inequality. This is because the purpose of health insurance systems is not to cater for trivial, cheap-to-treat ailments but for catastrophic, expensive-to-treat illnesses, affecting relatively few persons, compared to incomes. Hence, we would argue that a higher degree of inequality is built into the system but hope that presentation of our data would trigger future discussion on the topic.

(iii) *Reconciling the needs of the few against the needs of the many:* The considerations and analyses about allocative efficiency and fairness presented above could be perceived as pitting the needs of the few against the needs of the many (Leufkens et al., 2022). Is there a way to reconcile these competing objectives and create a win-win scenario?

We recall that orphan drugs have often been at the cutting edge of development in the domains of basic and pharmaceutical sciences, methodologies for evidence generation, and policy implementation. We see underutilised opportunities to create learnings at several levels from the use of (high-price) orphan drugs that can eventually benefit patients with non-orphan diseases.

Many new orphan products are and will be based on platform technologies such as monoclonal antibodies, protein replacement therapies, oligonucleotides, and gene and cell therapies (Tambuyzer, E. et al., 2020). One of the advantages of platform technologies is the opportunity of cross-product learnings about the manufacture and clinical use and performance of the technology. Although there may be important differences between each specific therapy, all members of a given platform share similar components.

Consider the example of chimeric antigen receptor (CAR) engineering technologies. Currently authorised CAR T cell therapies are difficult to manufacture and indicated only for hematologic malignancies, most of which are designated orphan conditions. Research in the field is continuing at a swift pace, including using T cells collected not from patients, but from healthy donors, and not only T cells but natural killer (NK) cells; there is reasonable hope that the CAR technology can be put to use in solid tumours, affecting much larger numbers of patients than can currently benefit from these products (National Cancer Institute and CAR). The ongoing research is informed by the post-marketing experience (National Cancer Institute, 2022) with current (orphan) CAR T cell products, including information on optimisation of manufacture, management of severe adverse effects, and optimal selection of patients (National Cancer Institute and CAR).

Another example of platform (National Cancer Institute, 2022) technologies characterised by cross-product and cross-indiation learnings is afforded by the broad field of oligonucleotide-based drugs; many of these products are first being developed for orphan diseases (Tambuyzer, E. et al., 2020). Yet, the on-market experience gleaned is now benefitting the development and utilisation of non-orphan drugs serving a wider group of patients.

Orphan drugs have also served as a testing ground for novel ways of evidence generation, such as the use of external control groups where RCTs are not feasible, or for implementation of new policies, such as regulatory accelerated pathways or pay-for-performance schemes.

The potential learnings from the development, reimbursement and utilisation of orphan drugs could be considerable and patients with non-orphan diseases could profit from the early treatment experiences with novel orphan drugs. Conceptually, this is a way in which patients with orphan diseases could “give-back” to the wider community of insured persons who foot their drug bill.

It is difficult to quantify the value of such learnings and we are not proposing to formally account for learnings during P&R negotiations of orphan drugs but it is the authors’ belief that every effort should be made to ensure maximum knowledge generation from every reimbursement contract covering high-price orphan drugs. The clinical outcomes of orphan drug treatment should be considered a “commons” and we would argue that, for example, pay-for-performance or other managed entry agreements (MEA) that keep confidential clinical outcome data are not just a wasted opportunity but are ethically unacceptable (Eichler et al., 2022).

There is one caveat, though: learnings can only materialise if all stakeholders contribute. Patients must see a moral obligation to make their experiences and data available for future learnings and consent to the secondary use of their data (under conditions of robust data and privacy protection), healthcare professionals need to make the effort to generate reliable clinical data, and manufacturers have to overcome their urge to keep learnings a secret.

Given the high stakes, we consider this a reasonable contribution to mitigate allocative inefficiency and support fairness of distribution.

#### Technical inefficiency

We now turn to addressing technical inefficiencies when paying for high-price orphan drugs. As discussed above, at a given price point, technical inefficiency is to a large extent a consequence of uncertainty.

(i) *Performance-based managed entry agreements:* Conceptually, performance-based managed entry agreements (PB-MEAs, also known as performance-based agreements, outcomes-based contracts, or pay-for-performance) could help to de-risk the economic consequences of clinical uncertainty by making some or all of the payment for a treatment contingent on the degree of patient benefit. Such a model could take several forms, with sliding scale bonuses or refunds depending on outcomes or instalments depending on duration of effect (For an in-depth discussion of PB-MEAs see Wenzl and Chapman, 2019).

While attractive in theory, critics argue that PB-MEAs increase administrative burden, reduce transparency, and that anticipated results are often not forthcoming or difficult to interpret (Wenzl and Chapman, 2019). The uptake of PB-MEAs by payers and manufacturers has been slow. There has been an increasing trend of relying on financial-based instead of outcomes-based MEAs because they are simpler and easier to implement, despite the fact that they can only mitigate some types of uncertainty, (e.g., around the size of the treatment population to limit budget impact) but do not de-risk uncertainties about the drug effect itself (e.g., size or persistence of effect) (Whittal et al., 2022). We argue that reluctance by payers or manufacturers to implement PB-MEAs is no longer an option for high-price, high-uncertainty orphan drugs.

Selection criteria to determine what products are considered “worthy” of PB-MEAs (Eichler et al., 2021) and negotiation frameworks for PB-MEAs (Whittal et al., 2022) have been developed, as have standards for collecting, analysing, and reporting of real world data (Orsini et al., 2020) and quality standards for disease registries (EMA, 2021). Hence, the tool-box for PB-MEAs is ready and the time may have come to require this kind of contract be applied for new orphan drugs (Pearson et al., 2022).

(ii) *Cross payer collaboration:* Yet, many payers are sceptical that they have the negotiating leverage to get manufacturers to agree to PB-MEAs (Pearson et al., 2022). Collaboration across payers will likely be required to ensure sufficient leverage and to yield meaningful financial savings as well as actionable real world evidence about orphan drugs (Pearson et al., 2022). EURORDIS, the European organisation for patients with rare diseases, have recently sketched out and published a proposal for the establishment of an EU-Fund to help finance access to transformative and potentially curative gene and cell therapies for very rare diseases. The fund should also co-finance the generation of post-marketing authorisation evidence across EU Member States during the years initially following approval, in order to reduce impact of uncertainties and enable joint price negotiations whilst at the same time allowing for timely access to life saving therapies (EURORDIS, 2021). While details of such a complex collaborative effort may still need to be hammered out, we consider the proposal a welcome step in the right direction. Whatever *modus operandi* can be developed by a given payer, we submit that implementation of new payment models and cross-payer collaboration will become inevitable tools to reduce technical inefficiencies in orphan drug reimbursement.

A collaboration of payers could also address the contentious issue of transparency of (production) costs, negotiated prices and rebates granted by manufacturers to individual payers. At present, such rebates are most often a tightly held secret. Some stakeholders argue that confidential rebates help bring down prices, others argue the opposite. We are not aware of any publicised, generalizable experience but would argue that the topic should best be broached by payer collaboratives.

## Discussion

Orphan drugs are fast entering the mainstream of healthcare systems. Given their own exigencies, including moderate effect sizes, high uncertainty, and high prices, business as usual is no longer an option for healthcare payers.

We have presented recommendations that may help healthcare payers address allocative and technical inefficiencies when reimbursing for high-price, high-uncertainty orphan drugs.

• Insist on financial transparency from a manufacturer with the aim of avoiding prices that would result in higher than industry average returns on investment
• Estimate the position of the anticipated cost/person/year on a Lorenz curve of drug spending in their own healthcare system with the aim of triggering additional action for any given product that would sit above a predefined spending threshold
• Ensure that learnings from orphan drugs including RWD generated become publicly available with the aim of benefiting patients with other, non-orphan diseases
• Accept or insist on PB-MEAs with the aim of de-risking the economic consequences of clinical uncertainty
• Seek collaboration across payers with the aim of maximising their negotiating leverage to get manufacturers to agree to PB-MEAs, and to yield meaningful financial savings as well as actionable real world evidence about orphan drugs.

We have not discussed here a range of additional proposals, including improved cross-country collaboration on HTA, and adaptive pathways to market access, that were recently proposed by patient and industry organisations to address patient access to medicines for rare diseases.

Also, our deliberations were focused on High-Income Countries. Yet, Low and Middle Income Countries (LMIC) are faced with the same challenges but have less funding available. Without being able to discuss in detail all issues of LMIC, we believe that the recommendations presented above are a good starting point if they can be combined with some form of Equity-Based Tiered Pricing, also known as international differential pricing (EFPIA-EURORDIS).

We are hopeful that a combination of the tools and considerations proposed may help balance the competing goals of stimulating orphan drug development while ensuring equitable access to drugs and sustainability of healthcare budgets.

## References

[B1] Aiuti A. Roncarolo M. G. Naldini L. (2017). Gene therapy for ADA‐SCID, the first marketing approval of an *ex vivo* gene therapy in europe: Paving the road for the next generation of advanced therapy medicinal products. EMBO Mol. Med. 9 (6), 737–740. 10.15252/emmm.201707573 28396566 PMC5452047

[B2] Berdud Mikel Drummond Michael Adrian Towse (2020). Establishing a reasonable price for an orphan drug. Establishing a Reason. price orphan drug Cost Ef Resour Alloc 18, 31. 10.1186/s12962-020-00223-x PMC7472708 32908456

[B3] EFPIA EURORDIS website EFPIA-EURORDIS joint statement on patient access to medicines for rare diseases. http://download2.eurordis.org/positionpapers/EFPIA_Eurordis_Statement_FINAL.pdf.

[B4] Eichler H.-G. Trusheim M. Schwarzer-Daum B. Larholt K. Zeitlinger M. Brunninger M. (2022). Precision reimbursement for precision medicine: Using real-world evidence to evolve from trial-and-project to track-and-pay to learn-and-predict. Clinical Pharmacology and Therapeutics 111 (1). 10.1002/cpt.2471 PMC9299639 34716918

[B5] Eichler H. G. Adams R. Andreassen E. Arlett P. van de Casteele M. Chapman S. J. (2021). Exploring the opportunities for alignment of regulatory postauthorization requirements and data required for performance-based managed entry agreements. International Journal of Technology Assessment in Health Care 37 (1), e83. 10.1017/s026646232100057x 34424152

[B6] EMA (2021). Guideline on registry-based studies. https://www.ema.europa.eu/en/documents/scientific-guideline/guideline-registry-based-studies_en-0.pdf.

[B7] EMA (2022). Committee for advanced therapies (CAT) https://www.ema.europa.eu/en/documents/minutes/minutes-cat-meeting-11-13-april-2022_en.pdf.

[B8] EURORDIS (2021). Access to affordable medicines for rare diseases – towards an equitable European ecosystem. https://download2.eurordis.org/ertc/ertc33/EUFRD%20reflection%20paper%2009112021_V9.pdf.,

[B9] Kohn D. B. Booth C. Shaw K. L. Xu-Bayford J. Garabedian E. Trevisan V. (2021). Autologous *ex vivo* lentiviral gene therapy for adenosine deaminase deficiency. The New England Journal of Medicine 384, 2002–2013. 10.1056/NEJMoa2027675 33974366 PMC8240285

[B10] Leufkens H. G. Kusynova Z. Aitken M. Hoekman J. Stolk P. Klein K. (2022). Four scenarios for the future of medicines and social policy in 2030. Drug Discovery Today 27, 2252–2260. 10.1016/j.drudis.2022.03.018 35364271

[B11] Lopez J. Banerji U. (2017). Combine and conquer: Challenges for targeted therapy combinations in early phase trials. Nature Reviews Clinical Oncology 14, 57–66. 10.1038/nrclinonc.2016.96 PMC6135233 27377132

[B12] National Cancer Institute (2022). CAR T cells: Engineering patients’ immune cells to treat their cancers. https://www.cancer.gov/about-cancer/treatment/research/car-t-cells (Accessed March 10, 2022).

[B13] Office for National Statistics The Gini coefficient. https://www.ons.gov.uk/peoplepopulationandcommunity/birthsdeathsandmarriages/families/methodologies/theginicoefficient#:∼:text=The%20Gini%20coefficient%20is%20the,of%20complete%20equality%20and%20inequality.

[B14] Official Journal of the European Communities (2000). (EC) No 141/2000 OF THE EUROPEAN PARLIAMENT AND OF THE COUNCIL of 16 December 1999 on orphan medicinal products. https://eur-lex.europa.eu/legal-content/EN/TXT/PDF/?uri=CELEX:32000R0141&from=EN.

[B15] Orsini L. S. Monz B. Mullins C. D. Van Brunt D. Daniel G. Eichler H. G. (2020). Improving transparency to build trust in real-world secondary data studies for hypothesis testing-why, what, and how: Recommendations and a road map from the real-world evidence transparency initiative. Pharmacoepidemiology and Drug Safety 29 (11), 1504–1513. 10.1002/pds.5079 32924243

[B16] Palmer S. Torgerson D. J. (1999). Economic notes: Definitions of efficiency. BMJ 318 (7191), 1136. 10.1136/bmj.318.7191.1136 10213735 PMC1115526

[B17] Pearson C. Lindsey S. Steven D. P. (2022). White paper - the next generation of rare disease drug policy: Ensuring both innovation and affordability. Boston, MA, USA: Institute for Clinical and Economic Review. 10.2217/cer-2022-0120 35946484

[B18] Tambuyzer E. Vandendriessche B. Austin C. P. Brooks P. J. Larsson K. Miller Needleman K. I. (2020). Therapies for rare diseases: Therapeutic modalities, progress and challenges ahead. Nature Reviews Drug Discovery 19 (2), 93–111. 10.1038/s41573-019-0049-9 31836861

[B19] Thomas K. Abelson R. (2019). The $6 million drug claim. New York, NY, USA: The New York Times.

[B20] Trusheim M. R. Berndt E. R. Douglas F. L. (2007). Stratified medicine: Strategic and economic implications of combining drugs and clinical biomarkers. Nature Reviews Drug Discovery 66 (4), 287287–329393. 10.1038/nrd2251 17380152

[B21] Villa F. Filippo A. D. Pierantozzi A. Genazzani A. Addis A. Trifirò G. (2022). Orphan drug prices and epidemiology of rare diseases: A cross-sectional study in Italy in the years 2014-2019. Frontiers in Medicine 17, 820757. 10.3389/fmed.2022.820757 PMC8891228 35252257

[B22] Wenzl M. Chapman S. (2019). Performance-based managed entry agreements for new medicines in OECD countries and EU member states: How they work and possible improvements going forward. OECD health working papers. Paris, France: OECD Publishing. 10.1787/6e5e4c0f-en

[B23] Whittal A. Jommi C. De Pouvourville G. Taylor D. Annemans L. Schoonaert L. (2022). Facilitating [corrected] more efficient negotiations for innovative therapies: A value-based negotiation framework. International Journal of Technology Assessment in Health Care 38 (1), e231–e238. 10.1017/S0266462322000095 35274602

[B24] Yao S. Chen Z. Yu Y. Zhang N. Jiang H. Zhang G. (2021). Current pharmacological strategies for duchenne muscular dystrophy. Frontiers in Cell and Developmental Biology 9. 10.3389/fcell.2021.689533 PMC8417245 34490244

